# The Correlation between Residual Primitive Reflexes and Clock Reading Difficulties in School-Aged Children—A Pilot Study

**DOI:** 10.3390/ijerph20032322

**Published:** 2023-01-28

**Authors:** Agata Kalemba, Maria Lorent, Sally Goddard Blythe, Ewa Gieysztor

**Affiliations:** 1 Student Research Group of the Developmental Disorders of Children and Youth, Department of Physiotherapy, Faculty of Health Sciences, Wroclaw Medical University, 50-367 Wroclaw, Poland; 2 The Institute for Neuro-Physiological Psychology, Chester CH4 9BR, UK; 3 Laboratory of Clinical Bases of Physiotherapy, Department of Physiotherapy, Faculty of Health Sciences, Wroclaw Medical University, 50-367 Wroclaw, Poland

**Keywords:** children, primitive reflexes, clock, dyscalculia, developmental disorders, neuromotor development

## Abstract

The aim of the pilot project was to research relationships between the occurrence and level of intensity of primitive reflexes in primary school children, the ability to read an analogue clock and to tell the time. A group of 28 children (14 girls and 14 boys) who attended Montessori Primary School was examined. In the first stage, participants were assessed for the presence of five primitive reflexes (PR): the asymmetrical tonic neck reflex (ATNR), symmetrical tonic neck reflex (STNR), spinal Galant reflex, tonic labyrinthine reflex (TLR) and Palmar grasp reflex. Romberg’s test was employed to identify signs of difficulties with control of balance and/or proprioception. In the second stage, pupils underwent tests that challenged their ability to read a clock and calculate passing time. After summing up points obtained for all tests, a correlation coefficient was made from which the results were derived. There is a negative correlation between the ability to read an analogue clock and the continued presence of some primitive reflexes. Lower neuromotor maturity (higher points of PR) correlates with lower ability to read a clock. The highest correlations between difficulty with telling the time were found with persistence of the STNR, ATNR and Romberg’s test.

## 1. Introduction

Primitive reflexes (PR) are an important index of children’s neuromotor maturity. They are examined after birth when their relative strength indicates normal development of the central nervous system (CNS). The reflex reactions are linked to early spontaneous movements and children’s psychomotor development is closely linked to the sequenced time of activity, inhibition and integration of individual reflexes. Brain stimulation from sensors and movement activity combined with the normal process of maturation facilitates the appropriate development of different stages of growth [1]. 

Primitive reflexes emerge from 9–12 weeks after conception and are observed in babies born at full term (40 weeks) [1,2,3,4]. They are mediated in the brain stem. Between the 6th and 12th month as a result of maturation of the central nervous system, their activity gradually recedes as postural reflexes emerge alongside the development of more advanced motor activities [1]. Postural reflexes provide a foundation for further psychomotor development of the child. They support subconscious control of posture and balance [2], taking up to three years of age to be fully developed. A delay in occurrence of a particular reflex reflects immaturity in the functioning of the CNS and can interfere with the child’s psycho-motor development. The activity of primitive reflexes is closely linked to the later emergence of postural reflexes and vice-versa, which in turn can interfere with a child’s ability to interact physically with the environment [5]. 

Gradually, as primitive reflex response is superseded by postural reflexes and increased voluntary control of posture, balance and coordination, psycho-social, motoric, educational skills and aspects of emotional development, which depend on postural control to provide a secure physical reference point in space, should be observed. The research on primitive reflexes shows that, in preschool or school-age children, they may still persist, especially in children in whom some spheres of development are disturbed [6,7,8,9,10,11]. Children with persistent PR are seen to have lower motor, cognitive, visual and learning achievements [12,13,14]. Some of the research shows specific difficulties in reading [15,16,17,18] or language disorder [8] in relation to PR activity. Moreover, primitive reflexes have been shown to persist in children of school-age who have specific learning difficulties [19,20], with the asymmetrical tonic neck reflex (ATNR) and symmetrical tonic neck reflex (STNR) being particularly prevalent amongst this group, followed by the Palmar grasp reflex and tonic labyrinthine reflex (TLR). Difficulty performing the Romberg test is also of significance.

The ability to tell the time develops in preschool (in countries where formal schooling starts from 6 years of age) and this skill is used throughout the whole of life [21], so being able to read a clock or estimate time is an important goal in primary school [22]. The ability to perceive time is a complex cognitive competence based on literacy, memory, arithmetic and spatial skills [23].

PRs under examination in this project are those which have been shown to be linked to other aspects of child development and to specific learning difficulties. The ATNR and STNR, for example, are elicited as a result of lateral rotation of the head (ATNR) or flexion and extension of the head (STNR). Both head movements provide stimulation to the vestibular system, but the reflex response allows only a limited and stereotyped reaction in terms of adjustment of muscle tone and its effect upon posture. Signs of difficulty in performing Romberg’s test may be secondary to an underlying conflict between vestibular function, muscular reaction and proprioceptive feedback, affecting physical stability and realisation of position in space. A secure reference point in space is an important precursor to being able to orientate in space and understand directional and spatial relationships and is fundamental to some of the spatial cognitive processes involved in reading an analogue clock or many other mathematical competencies (e.g., arithmetic abilities).

Levinson suggested that underlying directional disturbances are a functional problem of the “inner ear’s compass” [24]. Vertical stability in space, of which postural control is both instrumental and an outcome, is essential for being able to understand spatial relationships such as the ability to differentiate left from right, up from down, etc., abilities that are basic to being able to understand the meaning of the upper and lower sections of a clock face and that left and right sides of a clock indicating before and after. Spatial deficits, which may be a secondary consequence of immature postural control, can potentially affect higher cognitive abilities such as directional awareness, misinterpretation of numbers, meaning derived from which hand is pointing at the numbers and understanding of clockwise movements.

Analogical reasoning in humans plays a prominent role in problem solving and involves application of past experience to new but similar experience based on physical knowledge of the world. Retained or residual primitive reflexes in school-aged children may contribute to, or be a reflection of, disassociation in the functional relationship between the vestibular and proprioceptive systems affecting equilibrium, which is chiefly related to space and supports cognitive understanding of spatial relationships.

The sense of physical stability in space involves both corporal space—that which is sensed as a result of information to and inside the body—and outside space, from which the basic notion of body schema (the body’s representation at the level of the cortex) is obtained through the experience of movement and the motoric adaptations to outside space. Partial retention of primitive reflexes in children of school age signifies immaturity in the acquisition of vertical stability in space, of which difficulty in performing the Romberg test is only one sign.

Furthermore, the ATNR divides the body into two parts, left and right, and similarly the STNR into upper and lower. The clock must be interpreted by a child in terms of sections describing halves, quarters and minutes as well as the understanding of clockwise motion.

Observing that the occurrence of persistent primitive reflexes has a direct connection to learning disorders we would like to answer two questions:

1. Is there a connection between persistence of primitive reflexes and the ability to read the time from an analogue clock or to calculate passing time?
2. Which of the reflexes have the most significant impact on the ability to read a clock?

## 2. Materials and Methods

This study is a part of the *Primitive Reflexes and All Children Sphere (PRACS) Project*, the aim of which was to determine researching reflexes and their influence on motor, sensory and cognitive development in preschool and school-aged children. The research has been certified by the Bioethical Committee of the Wrocław Medical University, no. of permit: KB-626/2018.

In a group of primary school children, the incidence of selected primitive reflexes was tested. Every parent signed a consent form for their child to take part in the research. They were also informed about the aim and the non-invasive character of the research.

### 2.1. Group Characteristics

Twenty-eight children took part in the research: 14 boys (50%) and 14 girls (50%) who attended the Montessori Primary School. The average age of the group was 8.14 (Me = 8). 

### 2.2. Testing the Activity of Primitive Reflexes

The children were tested for the following reflexes: ATNR quadruped test (Ayres), ATNR Hoff-Schilder test, STNR, Spinal Galant, TLR and Palmar grasp reflex. They also undertook Romberg’s test. Points were given depending on the level of activity of a reflex. The tests were administered as in the INPP screening test [25], where a score of 0 described no reflex (no abnormality detected) and 4 meant that the reflex was fully retained. The scores have been averaged for each reflex, which were tested in two positions, e.g., left and right, or flexion and extension.

Tests for the ATNR using the Hoff-Schilder test and TLR were carried out with the participant’s eyes closed. Romberg’s test was administered first with the eyes open, then with the eyes closed. ATNR Ayres, STNR and GALANT reflexes were carried out with the test subject in the quadruped position with the eyes open. ATNR Hoff-Schilder, TLR, Palmar grasp reflex and Romberg’s test were conducted in the erect position.

The Asymmetrical Tonic Neck Reflex (ATNR) was assessed in two ways. Firstly, it was observed whether passive lateral rotation of the head to either side in the quadruped position was accompanied by involuntary flexion in the upper occipital limb (bending of the cubital and glenohumeral joints), movement of the torso, or pelvis. Secondly (ATNR Hoff-Schilder test), each child was asked to stand with their feet together, arms extended to the front and wrists floppy. The researcher turned the child’s head in both directions. The test was deemed positive when one or both of the arms followed the movement of the head and there was no isolation of head movement from the rest of the body. 

For the STNR, the researcher instructed the child in the quadruped position to: “extend your head as if looking towards the ceiling” and then, “flex your head as if looking between your knees”. During the extension phase, the focus was on any increase in extensor tone in the arms and flexion in the lower body such as tendency to sit on the heels. During the flexion phase, a positive score was recorded if there was increased flexor tone in the arms and extensor tone in the lower body.

Assessment of the TLR involved the child actively flexing and extending the head while in the erect position with the feet together. During the flexion phase, any increase in flexor tone throughout the length of the body was observed. During the extension phase, compensations such as increased extensor tone in the legs or other forms of upper limb movement, standing on tip-toes, or loss of balance indicated that the reflex is active.

The Palmar Grasp reflex (GRASP) and spinal Galant reflex are skin reflexes. Assessment of the Palmar reflex was made by applying gentle stimulation to the palm of the hand administered by the researcher. This was applied using the blunt end of a pencil as the stimulus. A positive response was detected when the child flexed finger II–V or there was adduction of the thumb. The test was repeated on both sides while simultaneously observing the symmetry of the reaction.

Tactile stimulation was also used to test the spinal Galant reflex. The researcher made a vertical movement with their thumb along the erector spinae muscle of the child on each side. When the reflex was active, a torso bending movement away from the stimulated side could be observed. 

During the Romberg test, the children were asked to keep their feet together while standing with the arms at the sides and to stand still with the eyes open. The test was then repeated with the eyes closed.

All the tests were completed by the clinicians with proper preparing and with 8 years of practice of examination and therapy of children with neuromotor immaturity. The results of the two independent clinicians were compared. The detailed form of the examination is described in Gieysztor et al. [9]. 

### 2.3. The Clock Test

The second part of the examination required participants to complete the clock test on their own. This part was administered by the teacher of the child. The test comprised seven tasks, four of which were open questions and three were choice tests. Each child could obtain a maximum of 23 points (100% correct answers). The questions assessed the ability to read the time on a clock shown in pictures and count the differences between hours.

### 2.4. Statistical Analysis

Test results of all the primitive reflexes assessed were tallied and, by summing up the results, an index of neuromotor status was obtained. The results of the clock test were summated and shown as a percentage. Statistical analysis was conducted using the IBM SPSS Statistics 25 version (IBM Corp., Armonk, NY, USA). The Pearson’s correlation coefficient was calculated. The established level of importance for analysis interpretation is *p* < 0.05.

## 3. Results

### 3.1. Descriptive Indices of the Activity Level of Reflexes and the Clock Test

An analysis of the level of activity of each reflex was conducted. The highest level of activity was observed in the ATNR (M = 1.8 points; SD = 1.06) and TLR (M = 1.1 p.; SD = 0.70). 

Calculating the CT points children obtained, the descriptive results shown average results of nearly half of the maximum points (M = 46%; SD = 0.35; Me = 29%). The descriptive statistics are shown in Table 1.

### 3.2. Correlation Analysis of the Clock Test Results and the Activity Level of Reflexes Results

An analysis of the correlation coefficient between the clock test (CT) results and the activity level of reflex test results was conducted. The results are shown in Table 2. There is a negative correlation between the results of the CT and the PR test (r = −0.34). That means that children with a lower score on the clock test had a higher incidence of persistent primitive reflexes (lower neuromaturation). Analyzing the correlation of the CT results with each reflex and looking for the answer to which have the most impact, we can see, that the STNR reflex shows a significant (*p* < 0.01) negative correlation with performance on the clock test. This means that the higher the level of activity in the STNR reflex observed in a child, the lower the results in the clock test. Tests for the ATNR, Palmar grasp reflex and Romberg’s sign also showed a statistically significant negative correlation with performance on the clock test (*p* < 0.05). The spinal Galant reflex and TLR showed a low negative correlation with performance on the clock tests.

### 3.3. Analysing the Specific Problems Observed in Children’s Answers

Analysing consistent errors made by children, we have indicated:

- Low ability to differentiate the functions of the different hands of the clock
- Misunderstanding the instruction for the task
- Lack of counting ability
- Ignorance of the nomenclature used in the context of the clock, i.e., not understanding the word ‘quarter’
- Lack of knowledge regarding reading full hours or difficulties in interpreting (answering 3:60 instead of 3:00, in case the short hand is located on 3 and long on 12)

## 4. Discussion

The aim of this study was to analyse the correlation between neuromotor development level, based on PR activity, and the ability to read a clock and to count passing time.

We have observed that children with lower neuromotor status had lower results in the clock test. Moreover, Pearson’s correlation shows that the most implicated reflexes were the STNR, followed by the ATNR, and soft signs of difficulties with balance and/or proprioception as revealed by the Romberg test. This is the first study, known to the authors, to compare neuromaturation status with the ability to read a clock and the results of tests to assess the ability to count passing time. 

However, the number of participants involved in the study was small. Although statistical analysis revealed trends in terms of higher incidence of PRs being associated with difficulty reading a clock or calculating passing time, further investigations are needed involving a larger numbers, which include control and comparison groups. 

To date, immature neuromotor status as identified by persistent primitive reflexes in children of school age has been shown to be elevated in children presenting with emotional and behavioural issues [26], children diagnosed with attention deficit hyperactive disorder (ADHD) [27], visual processing difficulties [28], immature motor skills [29,30], relation to motor skills in pre-school children [10], motor skills in relation to school readiness [31], communication disorder [32], developmental language disorder [8], differences in school performance [13,33] and social disadvantage [34]. This broader spectrum of issues found to be present in conjunction with signs of neuromotor immaturity suggests that it may be a general underlying factor in a number of developmental disorders and specific learning difficulties. In other words, immature neuromotor status may undermine performance in a range of “higher” cognitive skills and emotional regulation. 

Learning to read an analogue clock and count passing time involves multiple cognitive skills including, but not exclusive to: language, the ability to count from 1–12 (or 24) and understanding sequencing and spatial relationships. While several of these abilities may be involved in calculating time, the results of this study suggest that spatial problems linked to the retention of primitive reflexes, which can affect balance and postural control and therefore the physical basis for cognitive operations in space, may be of significance. These findings and the questions they raise could be used as a basis for a larger scale study to discover whether persistence of PRs is directly related to difficulty in learning to read an analogue clock and calculate passing time [2,25].

The results of our study confirm the hypothesis that some reflexes may play a special role in the ability to read an analogueclock. The highest coexistence of ATNR, STNR, or Romberg test with clock reading disturbances was the most important. Similarly, results suggest that subsequent areas of child development and related cognitive skills may be impacted by retention of primitive reflex activity, and point to potential areas of improvement after therapy for PR integration. Answering the question of why these particular reflexes have the most significant impact on the ability in reading the clock, we may think again about the function of underlying pathways that these reflexes and the Romberg test reflect. They are important for orientation in space and understanding directional and spatial relationships. Retained or residual primitive reflexes in school-aged children may lead to, or be a mirror of, inappropriate functional relationship between the vestibular and proprioceptive systems affecting equilibrium, which is particularly related to space and supports cognitive orientation of spatial relationships. Stability in space involves the stability of the body, based on the internal sensory information and environmental space, which is achievable through the experience of movement and the motoric adaptations to outside space.

So far, no study has been conducted, known to the authors, that proves the occurrence of persistent primitive reflexes in children who experience difficulties in reading the hour from a clock; however, some authors point to children who have problems with mathematics making numerous mistakes while trying to read the clock. Andersson et. al. and Burny et. al. suggest that difficulty in reading a clock can also be one of the first signals pointing to dyscalculia in early school-age children. They show that children who have mathematical difficulties also demonstrate increased challenges in clock reading [22,35,36]. Mutlu et al. show that the majority of children experience difficulty in not only reading the clock but also in correctly drawing the clock face and remembering the functions of the hands. The most common mistake shown in the research was paying attention to only one hand on the clock (minute or hour), which resulted in omitting the information about hour or minutes or incorrectly adding the missing element [37]. Similar problems were observed in our group of children with lower neuromotor maturation. We have observed a low ability to differentiate the functions of each hand of the clock, misunderstanding the instruction for the task, lack of counting ability, ignorance of the nomenclature used in the context of the clock, i.e., not understanding the word ‘quarter’, and lack of knowledge regarding reading full hours. Mutlu et al. claim that, while dyscalculia explains the cause of these difficulties in children at risk, no cause has been found for poor clock reading in children who have not been diagnosed with dyscalculia. Maybe in these cases lower neuro-maturity plays its role? To answer this question, wider research is necessary for investigating the abilities of clock reading in school-age children.

This pilot study presents neuromotor status as being a factor in being able to read a clock and calculate time; however, because of the small sample of participants, the claim should be treated as the very beginning. Looking at the limitations of the study, we have also found that it should be conducted with a larger population. It may also be expanded to investigate the role of lateralization as a foundation for being able to tell the time. Researchers may also look for other temporal aspects, using tests consisting of other spheres of time such as orientation, sequences, time units, clock reading, life span, birthday, or present time awareness.

## 5. Conclusions

1. Neuromotor maturity status may be related to the ability to read a clock and to count time.
2. There is a need to expand the research in this area on laterality and the research should be conducted with a bigger sample.
3. The recommendations to introduce reflex integration therapy to children with learning problems may also improve the ability to read a clock.

## Figures and Tables

**Table 1:** Descriptive statistics obtained in the examination of the level of reflexes and clock test performance.

	CT	PR	ATNR	STNR	Galant	ATNR H-S	Romberg	TLR	Grasp
M	0.46	1.14	1.80	0.88	0.73	1.20	0.26	1.10	0.38
SD	0.36	0.46	1.06	0.56	0.92	0.63	0.32	0.70	0.64
Me	0.29	1.00	1.50	1.00	0.50	1.00	0.00	1.00	0.00
Min	0.00	0.00	0.00	0.00	0.00	0.00	0.00	0.00	0.00
Max	1.00	3.00	4.00	2.00	3.00	2.50	1.00	2.50	2.50

M—mean; level of reflexes–neuromotor status, CT—clock test; PR—level of primitive reflexes; ATNR measured by Ayres test; STNR—symmetrical tonic neck reflex; ATNR H-S—asymmetrical tonic neck reflex measured by Hoff-Schilder test; TLR—tonic labyrinthine reflex.

**Table 2:** The results of correlation analyses performed for clock test and the activity level of reflexes.

	CT	PR	ATNR	STNR	Galant	ATNR H-S	Romberg	TLR	Grasp
CT	−	−0.34	−0.30	−0.6	−0.18	−0.26	−0.30	−0.15	−0.31
PR	−0.34	−	0.64	0.63	0.61	0.63	0.49	0.61	0.30
ATNR	−0.30	0.64	−	0.52	0.48	0.64	0.40	0.34	0.10
STNR	−0.60	0.63	0.52	−	0.15	0.51	0.28	0.32	0.27
Galant	−0.18	0.61	0.48	0.15	−	0.31	0.44	0.37	0.10
ATNR H-S	−0.26	0.63	0.64	0.51	0.31	−	0.49	0.38	0.16
Romberg	−0.30	0.49	0.40	0.28	0.44	0.49	−	0.59	0.02
TLR	−0.15	0.61	0.34	0.32	0.37	0.38	0.59	−	0.03
grasp	−0.31	0.30	0.10	0.27	0.11	0.16	0.02	0.03	−

CT—clock test; reflexes–summed points for reflexes; PR—primitive reflexes as a sum; ATNR measured by Ayres test; STNR—symmetrical tonic neck reflex; ATNR H-S—asymmetrical tonic neck reflex measured by Hoff-Schilder test; TLR—tonic labyrinthine reflex.

## Data Availability

Data can be provided upon request.
